# Factors Influencing the Discordancy Between Intraoperative Frozen Sections and Final Paraffin Pathologies in Ovarian Tumors

**DOI:** 10.3389/fonc.2021.694441

**Published:** 2021-07-01

**Authors:** Hung Shen, Heng-Cheng Hsu, Yi-Jou Tai, Kuan-Ting Kuo, Chia-Ying Wu, Yen-Ling Lai, Ying-Cheng Chiang, Yu-Li Chen, Wen-Fang Cheng

**Affiliations:** ^1^ Department of Obstetrics and Gynecology, College of Medicine, National Taiwan University, Taipei, Taiwan; ^2^ Department of Obstetrics and Gynecology, National Taiwan University Hospital, Xin-Chu, Taiwan; ^3^ Department and Graduate Institute of Pathology, College of Medicine, National Taiwan University, Taipei, Taiwan; ^4^ Graduate Institute of Clinical Medicine, College of Medicine, National Taiwan University, Taipei, Taiwan; ^5^ Graduate Institute of Oncology, College of Medicine, National Taiwan University, Taipei, Taiwan

**Keywords:** ovarian tumor, ovarian cancer, intraoperative frozen pathology, paraffin pathology, discordancy of diagnosis

## Abstract

**Aim:**

To retrospectively investigate the pre-operative clinical factors and ultrasonographic features that influence the accuracy of the intraoperative frozen section (IFS) of ovarian tumors.

**Patients and methods:**

Women with ovarian tumors that underwent IFS in one tertiary medical center were recruited from January 2010 to December 2018. Demographic and clinical data of these women were retrieved from medical records in the hospital’s centralized database.

**Results:**

A total of 903 ovarian tumors were enrolled, including 237 (26.2%) benign, 150 (16.6%) borderline tumor, and 516 (57.2%) malignant. The overall accuracy of IFS among all specimens was 89.9%. The sensitivities of IFS in diagnosing borderline tumors (82.0%) and malignant tumors (88.2%) were lower than in diagnosing benign tumors (98.7%, p <0.001, Z-test). The specificity of diagnosing malignant tumors (99.7%) was significantly higher than that of diagnosing benign tumors (94.7%, p <0.001, Z-test). The group with discordant IFS and final paraffin pathology (FPP) had younger age (47.2 ± 14.0 *vs.* 51.5 ± 11.8 years, p = 0.013, Mann–Whitney U test), and higher percentage of early-stage disease (85.2% *vs.* 65.1%, p = 0.001, chi-square test) and mucinous (39.3% *vs.* 3.3%) and endometrioid histologic types (34.4% *vs.* 20.2%) than the concordant group (all by chi-square test). Menopause (OR 0.34, 95% CI 0.15–0.76, p = 0.009), multicystic tumor in ultrasound (OR 2.14, 95% CI 1.14–4.01, p = 0.018), and ascites existence (OR 0.33, 95% CI 0.14–0.82, p = 0.016) were factors related to the discordant IFS by multivariate analysis.

**Conclusions:**

IFS has good accuracy in the diagnosis of ovarian tumors. We recommend more frozen tissue sampling for sonographic multicystic tumors in premenopausal women to improve the accuracy of IFS.

## Introduction

Ovarian tumors are common gynecologic problems occurring in females of all ages. In the United States, it is estimated that there is a 5 to 10% lifetime risk for women undergoing surgery for a suspected ovarian neoplasm (1, 2). Among ovarian tumors, those that are benign account for the majority (around 60%), followed by the 20–30% that are malignant and <10% that are borderline tumor (3). The incidence of ovarian cancer has increased over the last 30 years in Taiwan, with the latest incidence being 1,521 per 100,000 women (4). The histological distribution of malignant tumors in Taiwan differs from that in Western countries, with fewer serous type and more clear cell type tumors (4).

The components of benign ovarian tumors are variable and include epithelial, germ cell, and sex-cord stromal tumors; endometrioma; ovarian abscesses; and functional ovarian cysts. Borderline tumors account for a minor proportion of ovarian tumors. They mainly originate from epithelial cells (5). Malignant ovarian tumors include epithelial, germ cell, and sex-cord stromal tumors, and the other rare histologic types such as sarcoma (6). In current clinical management, the surgical treatment of benign tumors includes tumor excision (cystectomy) or oophorectomy (7). For the management of borderline tumors, surgical approaches including hysterectomy, bilateral salpingo-oophorectomy, omentectomy, and pelvic and para-aortic lymphadenectomy are recommended. A fertility-sparing approach is a common alternative, particularly for those who desire to preserve fertility (5). However, for the management of ovarian cancer, surgery usually involves hysterectomy, bilateral salpingo-oophorectomy, omentectomy, and pelvic and para-aortic lymphadenectomy (8). Because the treatment strategies and prognoses are much different for benign, borderline, and malignant ovarian tumors, accurate diagnosis is important.

Ovarian tumors often represent a diagnostic challenge, first because of the vague symptoms and presentation (8). There are some clinical tools that help to differentiate the nature of ovarian tumors before surgery, including serum tumor markers and imaging studies such as ultrasonography, computer-assisted tomography (CAT), magnetic resonance imaging (MRI), and positron emission tomography (PET) (8). CA-125 is a non-specific tumor marker but is the most commonly used marker in ovarian tumors, CA-125 is not useful in differentiating ovarian benign or malignant tumors, due to its low sensitivity and specificity. Its levels can increase due to other kinds of malignancies (e.g. breast, lung, colon, and pancreatic cancer) and benign diseases (e.g. endometriosis, pelvic inflammatory disease, and ovarian cysts) (8).

Gynecologic ultrasound plays an important role in the initial evaluation of an ovarian tumor. Ultrasound relies on morphologic features to distinguish between benign and malignant lesions. Some predictive models to differentiate ovarian benign and malignant tumors have been developed (7). The risk-of-malignancy index is one such scoring system currently recommended by many national guidelines (9). Another model, the International Ovarian Tumor Analysis (IOTA) simple ultrasound-based rules (“simple rules”) has also shown good accuracy (10). However, these tools have their limitations, such as operator-dependent performance (11). The IOTA rules have high sensitivity and specificity according to expert subjective impressions, but worse accuracy when assessed using objective, calculated tools (12).

Needle aspiration and image-guided core biopsy are widely used for a variety of the other non-ovarian tumors, such as breast cancer, without adverse impact on prognosis (13). However, needle aspiration or core biopsy is less common clinical practice in the diagnosis of ovarian tumors, because these two procedures pose the risk of spillage and possible upstaging of malignant lesions (13, 14).

The more specific the extent of operation performed, the more benefit the patient receives, with less morbidity. In addition to the pre-operative imaging study and tumor markers, intraoperative frozen section (IFS) as diagnostic tool can aid procedural decision-making. IFS has been developed for more than 100 years and was first demonstrated by Wilson (15). It has become routine practice in the treatment of complex ovarian tumors during surgery. The good accuracy of IFS, from 90 to 99%, has been proven in many studies (16–18). Although IFS can provide very important and helpful information for clinical practice during surgery, it still does not provide exactly the same results as the final paraffin pathology. Only a limited number of studies have evaluated clinical preoperative characteristics and imaging features as factors influencing the accuracy of IFS.

Here we aimed to retrospectively investigate the concordance between IFS and final paraffin pathology (FPP) and factors that might influence discordancy between them. We propose pre-operative clinical factors and ultrasonographic features that can be used to predict the risk of discordancy of IFS and FPP in order to improve the accuracy of the IFS of ovarian tumors.

## Materials and Methods

### Patient Population and Data Collection

Women with adnexal (ovarian) tumors that underwent intraoperative frozen section (IFS) in one tertiary medical center were recruited from January 2010 to December 2018. This study protocol was approved by the Institutional Review Board of the hospital. Demographic and clinical data of these women were retrieved from medical records in the hospital’s centralized database. These data included the age at diagnosis, parity, menopausal status, co-morbidity, operative method, level of pre-operative tumor markers, and pre-operative ultrasound characters, International Federation of Obstetrics and Gynecologic stage, tumor histology and grade, type of surgery, and types and cycles of chemotherapy. Tumor markers including CA-125 (IU/ml), CA19-9 (IU/ml), CEA (ng/ml), and alpha-fetal protein (αFP) (ng/ml) from sera were also assayed and recorded before surgery. A transvaginal and/or transabdominal ultrasound was also performed before surgery. The selection of ultrasound characters was according to the IOTA rules (10).

Patients whose specimens for the final pathology analysis were not of ovarian origin were excluded. The FPP of all ovarian tumors was also retrieved. The tumors were classified histologically according the World Health Organization (WHO) classification of ovarian neoplasms in 2009, 2014 and 2020 (19–22), and the stage of each malignant tumor was based on the FIGO (International Federation of Gynecology and Obstetrics) staging system (21). Each ovarian tumor of this study would be classified into one of three categories—benign, borderline or malignant according to WHO ovarian tumor classification (22). The malignant tumors included epithelial tumors, sex-cord stromal tumors, germ cell tumors or other rare malignancies. The borderline tumors included different histologic types of epithelial borderline tumors. The benign lesions included all benign tumors and other non-tumor lesions such as corpus luteum cyst.

The specimens for IFS were analyzed by the pathologist in the laboratory in an unfixed state right after the surgeon removed them from the patients. At least two sections of all specimens were designated for frozen pathology analysis by the pathologist. The IFS of these ovarian tumors only reported benign, borderline, or malignant tumors without definite histologic type. The reports of paraffin pathology included benign, borderline, and malignant tumors together with their respective histologic type. We defined the concordant result when the reports of IFS and FPP were the same (benign, borderline or malignancy). Otherwise was defined as discordant result.

### Statistical Analysis

Using standard statistical formulas, we calculated the overall accuracy, sensitivity, and specificity with the 95% confidence interval (CI) between IFS and FPP. These were calculated for the three categories—benign, borderline, and malignant. If the results of IFS and final paraffin pathology were the same, they were defined as concordant, and otherwise as discordant. The proportional Z test, one-way ANOVA, chi-square test, Kruskal–Wallis test, and Mann–Whitney U test were used for statistical analysis. The factors contributing to discordancy between IFS and FPP among the malignant tumors were further analyzed by univariate and multivariate Cox logistic regression analysis. A p value less than 0.05 was regarded as statistically significant. Statistical analyses were all performed using SPSS 22.0 for Windows (IBM, Armonk, New York, NY).

## Results

### Patients’ Characteristics From Intraoperative Frozen Section

A total of 903 patients were enrolled. The basic characteristics of the 903 women with ovarian tumors that underwent frozen pathology are shown in **Table 1**. The mean age was 49.5 ± 13.4 years old (range 7–88). There were 464 (51.4%) women menopause. The majority of the women (97.3%) underwent laparotomy. The median pre-operative CA-125 value of the 903 women was 94.9 IU/ml (32.5–423.7, 25th–75th percentiles).

**Table 1:** Basic characteristics of the 903 women with ovarian tumors that underwent frozen pathology.

Parameter	Patient number (%)
**Menopause**	
No	464 (51.4)
Yes	439 (48.6)
**Parity**	
0	324 (35.98)
≥1	579 (64.1)
Comorbidity*	
No	592 (65.6)
Yes	311 (34.4)
**Operation method**	
Laparoscope	24 (2.7)
Laparotomy	879 (97.3)
**Pre-operative tumor markers**	
CA-125 (n = 881, U/ml), median (25th–75th%)	94.9 (32.5–423.7)
CA19-9 (n = 387, U/ml), median (25th–75th%)	29.3 (8.0–133.9)
CEA (n = 372, ng/ml), median (25th–75th%)	1.5 (0.8–2.6)
αFP (n = 104, ng/m), median (25th–75th%)	2.5 (2.0–3.9)

*Including cardiovascular disease, liver disease, malignancy other than ovarian cancer, diabetes mellitus, peptic ulcer, chronic kidney disease.

### Ovarian Malignant Tumors Accounted for the Majority of Intraoperative Frozen Pathology Diagnoses

There were 237 (26.2%) benign ovarian tumors, 150 (16.6%) ovarian borderline tumors, and 516 (57.2%) ovarian malignant tumors. As shown in **Table 2**, 699 (77.4%) of 903 tumors were of epithelial origin, followed by 66 (7.0%) sex-cord stromal tumors and 51 (5.6%) germ cell tumors. Endometrioma, tubo-ovarian abscess, hemorrhagic cyst, and corpus luteum accounted for 8.4% (n = 76) of the 903 tumors. There were two specimens that the IFS failed to classify into benign, borderline, or malignant. We classified these two cases as undetermined.

**Table 2:** Final paraffin pathologic characteristics of the 903 ovarian tumors.

Parameter	Number (%)
Stage*	
I/II/III/IV	279 (30.9)/70 (8.0)/138 (15.3)/28 (3.1)
**Tumor size** (cm, mean ± SD)	11.7 ± 7.3
Benign lesions other than cystadenoma**	76 (8.4)
**Germ cell tumor**	51 (5.6)
Mature teratoma	32
Malignant germ cell tumor	19
**Sex cord stromal tumor**	66 (7.0)
Fibroma/Fibrothecoma	43
Granulosa cell tumor	19
Others***	4
**Epithelial tumor**	699 (77.4)
**Serous**	181
Benign (% in serous tumor)	10 (5.5)
Borderline (% in serous tumor)	44 (24.3)
Malignancy (% in serous tumor)	127 (70.2)
**Mucinous**	215
Benign (% in mucinous tumor)	73 (34.0)
Borderline (% in mucinous tumor)	103 (47.9)
Malignancy (% in mucinous tumor)	39 (18.1)
**Endometrioid**	115
Benign (% in endometrioid tumor)	0 (0)
Borderline (% in endometrioid tumor)	2 (1.7)
Malignancy (% in endometrioid tumor)	113 (98.3)
**Clear cell**	167
Benign (% in clear cell tumor)	0 (0)
Borderline (% in clear cell tumor)	1 (0.6)
Malignancy (% in clear cell tumor)	166 (99.4)
**Mixed types carcinoma** ^#^	18
**Undifferentiated carcinoma**	3
**Carcinosarcoma**	9
**Sarcoma**	2

*The stage was applied to malignant cases with FIGO stage in 2009; **including endometrioma, hemorrhagic cyst, tubo-ovarian abscess and corpus luteum; ***including one Sertoli–Leydig cell tumor, two thecomas, and two steroid cell tumor with not otherwise specified; ^#^including 10 endometrioid with clear cell tumors, five endometrioid with mucinous tumors, one tumor with endometrioid and serous types, and two tumors with three different histologic types.

As shown in **Table 2**, there were 19 (37.3%) malignant germ cell tumors; 21 (31.2%) ovarian malignant sex-cord stromal tumors, including 19 granulosa cell tumors; two Sertoli–Leydig cell tumors; and 466 (65.6%) epithelial ovarian tumors. Among the malignant ovarian tumors, 69.8% (127/181) were serous, 18.1% (39/215) were mucinous, 98.3% (113/115) were endometrioid, and 99.4% (166/167) had clear cell histology of epithelial origin.

### Intraoperative Frozen Section Had Better Sensitivity for Benign Ovarian Tumors and Higher Specificity for Malignant Ovarian Tumors

We further evaluated the accuracy of IFS. **Table 3** shows the comparison of IFS and FPP of all 903 ovarian tumors. There were 812 concordant and 89 discordant pairs. The overall accuracy of IFS among all specimens was 89.9%. Two cases were not determined as benign, borderline, or malignant *via* IFS; one was a benign mature teratoma and one was a malignant clear cell carcinoma.

**Table 3:** Comparison of the intraoperative frozen pathology and final paraffin pathology of the 903 ovarian tumors.

		Paraffin pathology, n			
		Benign	Borderline	Malignancy	Total
	Benign	**234**	26	9	269
	Borderline	2	**123**	51	176
Frozen pathology, n	Malignancy	0	1	**455**	456
	Undetermined	1	0	1	2
	Total	237	150	516	903

n, number.

In bold: the concordance between frozen pathology and paraffin pathology.

All results regarding the accuracy, sensitivity, and specificity of IFS are shown in **Table 4**. The diagnostic accuracy, sensitivity, and specificity were 95.8, 98.7, and 94.7%, respectively, for benign ovarian tumors. The diagnostic accuracy, sensitivity, and specificity of IFS were 91.1, 82.0, and 93.0% for ovarian borderline tumors. The diagnostic accuracy, sensitivity, and specificity of frozen sections were 93.1, 88.2, and 99.7% for malignancy. The sensitivities in diagnosing borderline tumors and malignant tumors were significantly lower than in diagnosing benign tumors. In contrast, the specificity of IFS in diagnosing malignant tumors was significantly higher than in diagnosing benign tumors.

**Table 4:** The diagnostic performance of intraoperative frozen pathology.

	Benign (%)	Borderline (%)	Malignancy (%)
	(95% CI)	(95% CI)	(95% CI)
Accuracy	95.8	91.1	93.1
Sensitivity	98.7 (97.3–100)	82.0* (75.8–88.1)	88.2* (85.3–90.9)
Specificity	94.7 (93.1–96.4)	93.0 (91.1–94.7)	99.7^┼^ (99.3–100.0)

CI, confidence interval; *comparisons between borderline or malignancy and benign tumor; p <0.001.

**^┼^**comparisons between malignancy and benign tumor, p <0.001, all statistical analyses performed by use of the proportional Z-test.

### The Group With Discordant IFS and FPP Differed From the Concordant Group in Their Clinical and Histopathologic Characteristics

We further analyzed the clinical and histopathological characteristics of concordant and discordant IFS and FPP in 516 malignant tumors. As shown in **Table 5**, the discordant group had younger age (47.2 ± 14.0 *vs.* 51.5 ± 11.8 years, p = 0.013, Mann–Whitney U test), a lower percentage of menopause (31.1% *vs.* 56.9%, p <0.001, chi-square test), and a higher percentage of early-stage tumors (85.2% *vs.* 65.1%, p = 0.001, chi-square test) than the concordant group. In addition, the discordant and concordant groups had different percentages of various histological types (p <0.001, Mann–Whitney U test). The discordant group had a higher percentage of mucinous (39.3% *vs.* 3.3%) and endometrioid (34.4% *vs.* 20.2%) types, as well as lower percentages of serous (6.6% *vs.* 27%) and clear cell (6.6% *vs.* 35.6%) types as compared with the concordant group. More than 50% (24/39) of the mucinous malignant tumors could not be correctly diagnosed by IFS, and a relatively high percentage (18.6%) of endometrioid malignant tumors were not correctly diagnosed by IFS. In contrast, only 3.1% (4/127) of serous and 2.4% of (4/166) clear cell malignant tumors were not diagnosed using IFS. The other factors, including parity, pre-operative tumor markers, CA-125, and tumor size did not differ between these two groups.

**Table 5:** Patient demographics and clinical and histopathological characteristics of concordant and discordant intraoperative frozen and final paraffin pathologies of 516 women and their malignant ovarian tumors.

	Concordant	Discordant	p
	(N = 455)	(N = 61)	
**Age, yrs,** (mean ± SD)	51.5 ± 11.8	47.2 ± 14.0	0.013*
**Parity**			0.89^#^
0	165 (36.4%)	21 (34.4%)	
≥1	290 (63.6%)	40 (65.6%)	
**Menopause**			<0.001^#^
No	200 (44.0%)	42 (68.9%)	
Yes	255 (56.0%)	19 (31.1%)	
**Pre-operative CA-125 (U/ml)**			0.15^&^
Median (25th–75th percentile)	221.9	98.4	
	(49.4–761.6)	(52.2–613.5)	
**Tumor size** (cm)	10.8 ± 4.7	13.8 ± 14.7	0.28*
Mean ± SD			
**Stage**			0.001^#^
Early (I/II)	296 (65.1%)	52 (85.2%)	
Advanced (III/IV)	159 (34.9%)	9 (14.8%)	
**Kinds of malignancy**			
Epithelial	423 (93.0%)	54 (88.5%)	0.20^#^
Non-epithelial	32 (7.0%)	7 (11.5%)	
**Histologic type**			
Serous	123 (27.0%)	4 (6.6%)	<0.001^++^
Mucinous	15 (3.3%)	24 (39.3%)	
Endometrioid	92 (20.2%)	21 (34.4%)	
Clear cell	162 (35.6%)	4 (6.6%)	
Others^@^	31 (6.8%)	1 (3.1%)	

N, number; yrs, years; SD, standard deviation; *one-way ANOVA; ^#^chi-square test; ^&^Kruskal–Wallis test; ^++^Mann–Whitney U test; ^@^including malignant mixed Müllerian tumor, mixed type carcinoma, adenosarcoma, and undifferentiated carcinoma.

Our results indicate that early-stage tumors in young pre-menopausal women, as well as mucinous and endometrioid malignant ovarian tumors, had higher incidences of discordancy between IFS and FPP.

### Factors Contributing to the Discordancy of IFS and FPP of Malignant Ovarian Tumors Identified by Use of a Cox Logistic Regression Analysis

Finally, we evaluated factors that could influence the discordance of IFS and FPP diagnoses of malignant ovarian tumors, using Cox logistic regression analysis. As shown in **Table 6**, menopause (OR 0.36, 95% CI 0.20–0.63, p <0.001), multicystic tumor on ultrasound (OR 2.27, 95% CI 1.22–4.23, p = 0.010), and ascites (OR 0.39, 95% CI 0.16–0.94, p = 0.032) were factors related to the discordant IFS by univariate analysis. These three factors were also identified as independent factors related to the discordant IFS in multivariate regression analysis (menopause, OR 0.34 95% CI 0.15–0.76, p = 0.009; multicystic lesion on ultrasound, OR 2.14, 95% CI 1.14–4.01, p = 0.018; ascites, OR 0.33, 95% CI 0.14–0.82, p = 0.016).

**Table 6:** Logistic regression analysis of clinical factors for discordancy between intra-operative frozen and final paraffin pathologies of 516 ovarian malignant tumors.

Parameter	Univariate		Multivariate	
	OR (95% CI)	p	OR (95% CI)	p
**Parity**				
0	Reference	0.73	Reference	0.12
≥1	1.11 (0.62–1.99)		1.68 (0.87–3.25)	
**Post-menopause**				
No	Reference	<0.001	Reference	0.009
Yes	0.36 (0.20–0.63)		0.34 (0.15–0.76)	
**Pre-operative CA-125 level (U/ml)**				
<35	Reference	0.29	Reference	0.39
≥35	1.56 (0.68–3.57)		1.43 (0.64–3.20)	
**Ultrasound finding Solid part**				
No	Reference	0.91	Reference	0.28
Yes	0.97 (0.56–1.69)		0.71 (0.38–1.32)	
**Multicyst**				
No	Reference	0.010	Reference	0.018
Yes	2.27 (1.22–4.23)		2.14 (1.14–4.01)	
**Tumor ≥10 cm**				
No	Reference	0.71	Reference	0.89
Yes	0.90 (0.52–1.57)		1.04 (0.59–1.82)	
**Intra-tumoral blood flow**				
No	Reference	0.20	Reference	0.069
Yes	1.45 (0.83–2.56)		1.79 (0.96–3.35)	
**Ascites**				
No	Reference	0.032	Reference	0.016
Yes	0.39 (0.16–0.94)		0.33 (0.14–0.82)	

OR, odds ratio; CI, confidence interval; yrs, years; SD, standard deviation.

Our results indicate that menopause and the existence of ascites were two associated factors for correct IFS diagnosis, whereas a multicystic lesion detected by ultrasound was a risk factor for wrong diagnosis from IFS.

## Discussion

Tumor markers and imaging studies are two preoperative assessment tools used to evaluate the nature of ovarian tumors. However, tumor markers can be elevated in many clinical situations, including benign and malignant conditions, and this results in lower specificity. Imaging studies include sonography, computer-assisted tomography, magnetic resonance imaging, and positron emission tomography. Gynecologic ultrasound helps differentiate ovarian tumors according to their morphology and echogenicity. Color Doppler ultrasound can provide more information with which to detect malignant tumors, by measuring intra-tumoral blood flow (10). Ultrasound is personnel dependent, and it is difficult to distinguish borderline tumors from malignant tumors even with the help of color Doppler ultrasound (23). The other imaging tools have good predictive rates in cancer staging but can be of limited use in differentiating the origins of ovarian tumors, especially in early stages (24). Most important is that these tools do not provide an exact diagnosis of benign, borderline, or malignant tumors, because of their macroscopic view. However, the microscopic view provided by IFS can offer a more concrete diagnosis and evidence that together aid the surgeon in planning the rest of the operation.

Surgeons depend on the results of intraoperative frozen pathology for decision making in dealing with ovarian neoplasia. Making a correct diagnosis of an ovarian tumor before surgery remains problematic. Ovarian tumors, unlike other malignancies, are seldom diagnosed *via* needle or punch biopsy, for several reasons. The greatest concern about ovarian tumor biopsy is that this procedure may result in tumor leakage or rupture, with subsequent intraperitoneal spreading of cancer cells if the tumor is malignant (13). Uncertain diagnoses can lead to the assumption of ovarian malignancy and thus create a risk of unnecessary surgery that can harm patients. Inadequate primary surgery can lead to a second operation and subsequent delay in treatment. Intraoperative frozen pathology improves decision making and management during the operation, avoiding unnecessary or delayed surgery (25). There are several indications for IFS including the tissue type, benign or malignant nature of the tissue, type of malignancy, determination of surgical margins, positivity of lymph nodes, and presence of malignant implants and/or metastases in other tissues or organ (26).

IFS showed excellent sensitivity and specificity in diagnosing ovarian malignancy. Many studies have evaluated the performance of IFS in single institutes, as in our study (16, 17, 27–29). The sensitivity in diagnosing malignant ovarian tumors ranged from 76 to 96%, with almost 100% specificity in diagnosing malignant tumors (16–18, 27). The meta-analysis by Cochrane Reviews revealed that IFS for the diagnosis of early-stage ovarian cancer has an average sensitivity of 90.0% (95% CI 87.6 to 92.0%) and specificity of 99.5% (95% CI 99.2 to 99.7%) (29). The diagnostic performance of this study was comparable to those of prior studies (16–18).

Histologic type was a confounding factor influencing the accuracy of IFS (28, 30). IFS is relatively inaccurate in diagnosing mucinous and borderline tumors (18). The sensitivity and specificity of IFS for borderline tumors were the lowest compared with those for benign and malignant ovarian tumors. The highest discordancy occurred when the IFS diagnosis was borderline tumors while the paraffin pathology indicated malignancy. The reported diagnostic performance of IFS for borderline tumors varies from study to study, with generally poor performance (31). The lowest accuracy and sensitivity of IFS in the diagnosis of borderline tumors was in the misdiagnosis of malignant tumors as borderline tumors. One of the reasons for this was the pathologist’s conservative attitude in reporting the IFS results. For borderline tumor, we also compared pre-operative clinical factors between the concordant and discordant groups of the borderline tumor, but none differed significantly between these two groups (data not shown).

Cancer stage was another factor that influenced the accuracy of IFS. There were more early-stage malignancies misdiagnosed by IFS than advanced staged malignancies. Clinical manifestations such as extra-ovarian spreading and intra-abdominal metastasis can provide more information for pathologists in the diagnosis of IFS (32). Early-stage malignancies and borderline tumors share gross morphological features. In this survey, more than 80% of the malignancies having discordant IFS and paraffin pathology were early-stage tumors. More malignant mucinous tumors in early stages were also found a confounding factor that influenced the IFS diagnosis.

Age was another confounding factor of the accuracy of IFS. The group with discordant IFS and FPP had younger age and lower percentage of menopause than the group with concordant results. Multivariate analysis identified menopausal status as an independent confounding factor affecting the discordancy of IFS and FPP. This was not revealed by prior IFS studies (16, 17, 33). Our explanation is that several correlations were noted between menopausal status and cancer stage or histologic type. There were 72.3% pre-menopausal patients with early-stage ovarian malignancy, in contrast to 63.1% menopausal patients (p = 0.03, chi-square test).

Preoperative clinical manifestation and sonographic features can help improve the accuracy of diagnosis using IFS. Our study also focused on clinical characteristics of ovarian tumors with discordancy of IFS and PPF has met an unmet clinical need. Prior studies have not identified any clinical or imaging features as risk factors for discordant IFS and paraffin pathology results (16, 17, 33). A multicystic lesion by preoperative sonography is an independent risk factor for IFS discrepancy in malignant tumors. We recommend increasing the number of sections examined in IFS when diagnosing multicystic tumors, in order to avoid and lower the rate of discordant IFS and FPP. Cimic et al. have also recommended using cytology to improve the accuracy of IFS (34). The presence of ascites by sonography reduced the risk of discordant IFS and FPP diagnoses of malignant ovarian tumors in this study. Ascites always occurred in advanced disease, so the presence of ascites in pre-operative sonography became an associated factor for the accuracy of IFP.

There were three cases in which the IFS showed more advanced results than the FPP. Two were cases in which IFS revealed mucinous borderline tumors while their FPP diagnosis was mucinous benign cystadenoma. These two cases had histologic features of borderline tumor in the IFS samplings. However, these features of borderline tumor accounted for less than 10% of the whole tumor area in paraffin sections (20). Thus, the pathologist changed the diagnosis of borderline tumor to benign cystadenoma. The IFS of the third case first revealed low-grade serous carcinoma and the FPP was changed to micropapillary serous borderline tumor, also called non-invasive low-grade serous carcinoma (20, 35). The low-grade serous carcinoma and micropapillary serous borderline tumor belong to the same spectrum of ovarian tumors, and it is difficult to differentiate them by IFS.

This study has several advantages. First, the pathologists’ experience and individual performance can influence the accuracy of IFS. Here, more than 90% of the IFS was reported by three well-experienced gynecologic pathologists in order to lower the individual bias of IFS. The diagnostic performance was comparable with that of prior studies (16, 18, 29). Second, our study provided a relatively large case number for analysis. Only one prior study recruited over 1,000 patients, and the other studies have analyzed fewer than 500 patients (18, 29). Moreover, we identified three pre-operative features—menopause, multicystic tumor, and ascites by ultrasonography—that help to reduce the discordancy between IFS and FPP.

## Conclusion

Our study demonstrated that IFS has good accuracy in distinguishing among benign, borderline, and malignant ovarian tumors. The discordancy of IFS was higher in younger, pre-menopausal patients, and for early-stage ovarian malignancies, especially the mucinous histologic type. Multicystic tumor in sonography was an independent risk factor of discordancy of IFS for malignant tumors. In contrast, menopause and ascites detected by sonography protected against discordancy. We recommend examining more frozen tissue samples for sonographic multicystic tumors in premenopausal women in order to avoid the wrong IFS diagnosis and to minimize the inappropriate surgical treatment of women with ovarian tumors.

## Data Availability Statement

The data analyzed in this study was obtained from hospital’s centralized database, the following licenses/restrictions apply: The dataset of this study can only be accessed under the permission of IRB. Requests to access these datasets should be directed to (wenfangcheng@yahoo.com).

## Ethics Statement

The studies involving human participants were reviewed and approved by Institutional Review Board of National Taiwan University Hospital. Written informed consent from the participants’ legal guardian/next of kin was not required to participate in this study in accordance with the national legislation and the institutional requirements.

## Author Contributions

Conception and design: W-FC, development of methodology: HS and W-FC. Acquisition of data (provided animals, acquired and managed patients, provided facilities, etc.): HS, H-CH, Y-JT, K-TK, C-YW, Y-LL, Y-CC, Y-LC, and W-FC. Analysis and interpretation of data (e.g., statistical analysis, biostatistics, computational analysis): HS, Y-JT, Y-CC, Y-LC, and W-FC. Manuscript writing: HS and W-FC. Review, and/or revision of the manuscript: YJ-T, C-YW, Y-LL, Y-CC, and Y-LC. All authors contributed to the article and approved the submitted version.

## Conflict of Interest

The authors declare that the research was conducted in the absence of any commercial or financial relationships that could be construed as a potential conflict of interest.
